# Use of Coronary CTA to Triage Patients With Low to Intermediate Risk for CADs in an Acute Care Facility Can Help Lower Healthcare Costs When Compared With the Current Standard of Care: A Retrospective Study

**DOI:** 10.7759/cureus.77962

**Published:** 2025-01-25

**Authors:** Olaniyi Fadeyi, Saviz Saghari, Varun Dang, Abhirami Shankar, Harpreet Singh

**Affiliations:** 1 Internal Medicine, West Anaheim Medical Center, Anaheim, USA; 2 Cardiology, West Anaheim Medical Center, Anaheim, USA

**Keywords:** cad: coronary artery disease, coronary computed tomoangiography, cost of hospitalization, healthcare cost savings, low-cost healthcare, retrospective studies

## Abstract

Acute chest pain is one of the most common reasons for ED visits in the United States. Most patients are eventually admitted to the hospital to “rule out ACS” even when there are no significant EKG abnormalities or elevated cardiac enzymes. In addition to undergoing expensive tests while in the hospital, patients are also exposed to iatrogenic harm thereby worsening the overall healthcare costs. Meanwhile, the use of coronary computed tomography angiography (CTA) as a “gatekeeper” diagnostic test for patients with low to intermediate risk for coronary artery diseases (CADs) has significantly lowered hospital admissions and associated costs. However, coronary CTA may not be helpful for all classes of patients. Therefore, this study seeks to determine if the distribution of patients presenting to the ED with chest pain in an acute care facility will justify an investment in coronary CTA and contribute to lowering healthcare costs. Patients’ data between July 2022 and June 2023 were considered in our analysis. Results revealed that a significant number of patients who presented to the ED for chest pain and were subsequently admitted to the hospital for further work-up would have benefited from coronary CTA screening without any need for further inpatient work-up. Also, cost analysis showed that the use of coronary CTA would have helped significantly lower healthcare costs in this facility.

## Introduction

Acute chest pain (ACP) is one of the most common triggers for ED visits in the United States [1]. It accounts for almost seven million annual ED visits with an associated cost of $13 to $15 billion [2]. Several patients who present to the ED with complaints of ACP undergo unnecessary tests and subsequent admission to the hospital thereby worsening the healthcare costs [3]. Within the context of medicolegal implications of discharging ACP patients who may be at risk for acute coronary syndrome (ACS), a safeguard option to admit such patients to “rule out ACS” is mostly preferred despite the cost implications [4]. The disadvantages of this practice include iatrogenic complications, increased probability of false positive results, and added expense [4]. Meanwhile, recent technological advances have shown that coronary computed tomography angiography (CTA) can be safely deployed to rule out coronary artery diseases (CADs) in patients with low to intermediate risk [5]. It was established in previous studies that coronary CTA has a high negative predictive value for diagnosing CADs which makes it very effective at lowering hospital admissions and thereby preventing unnecessary coronary angiography tests during work-up [6]. Recent studies have shown that coronary CTA has a sensitivity of 94-99% for the diagnosis of CADs [7]. In fact, European Society of Cardiology (ESC) recommends coronary CTA as the first-line option to exclude CADs in patients with low to intermediate risk [8]. It is against this backdrop that coronary CTA has become the mainstay diagnostic tool for the assessment of coronary artery atherosclerosis. Therefore, this study seeks to evaluate the characteristics of patients admitted into an acute care facility for ACP between July 2022 and June 2023 to determine if the facility has a significant population of ACP patients in the low- to intermediate-risk bracket to justify an investment in coronary CTA when compared with the current standard of care.

## Materials and methods

**Figure 1:**
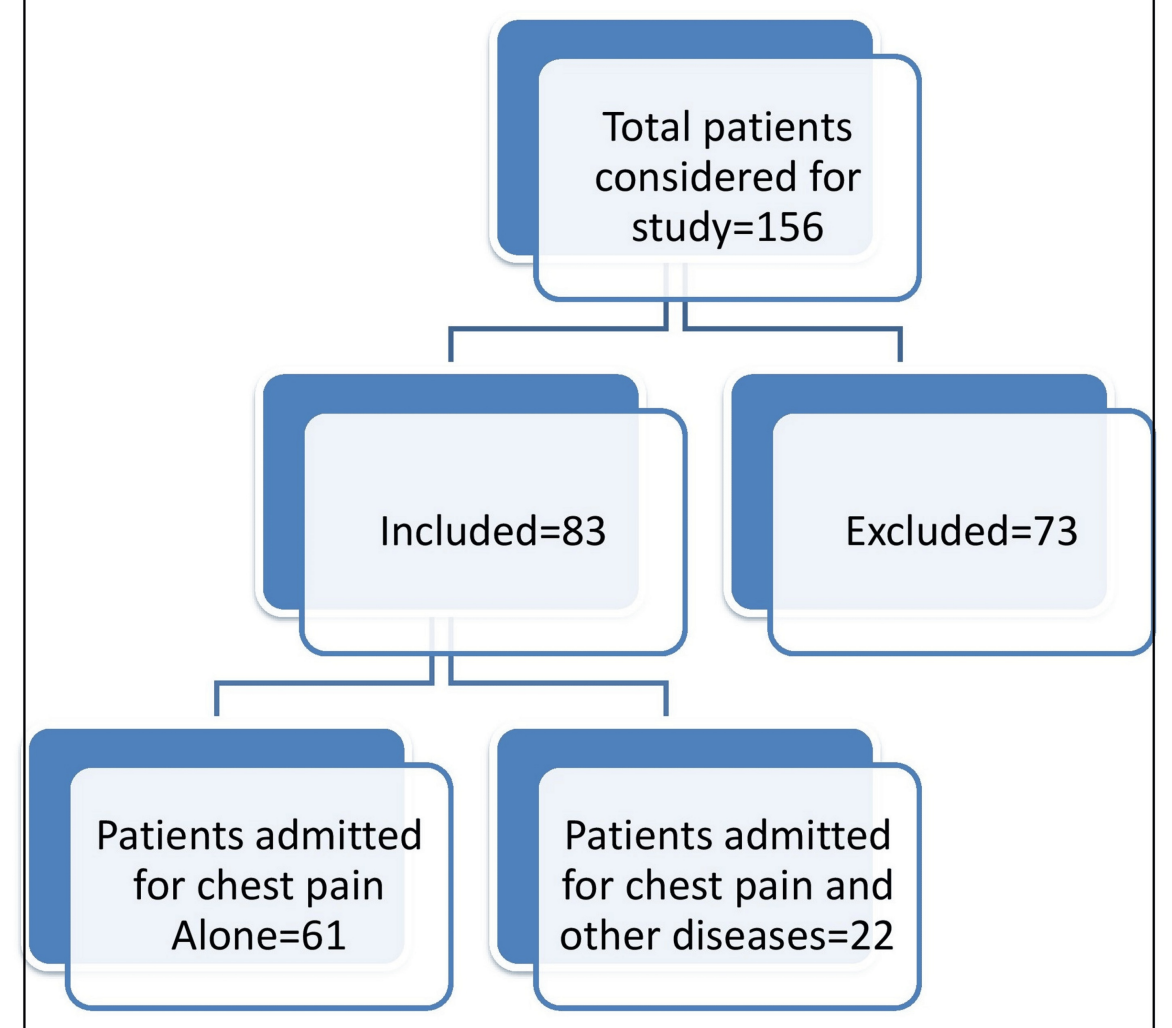
Distribution of patients considered for this study

This research project was reviewed and approved by the institutional review board (IRB) of California University of Science and Medicine. The project site is a 219-bed acute care hospital (West Anaheim Medical Center, Anaheim) located in California. The target population is patients who were admitted into the hospital for ACP between July 2022 and June 2023. This is a retrospective study. Patients’ information was retrieved from the electronic medical record. Patients with a history of CADs with coronary artery stents placement, CABG, prior cardiac surgery, structural heart disease, congenital heart disease, severe CKD, arrhythmias, obesity, contraindication to beta blocker use, and elevated troponin triggered by ongoing chest pain were excluded from the study while patients presenting with ACP without marked electrocardiogram abnormalities, elevated cardiac enzymes and history of heart disease were considered. Furthermore, patients who met the inclusion criteria but presented with other conditions that warrant inpatient management regardless of the outcome of coronary CTA results were equally exempted from the study. There were no restrictions based on age or gender. Care trajectory including further inpatient work-up and clinical outcomes were followed and documented. For each patient, attention was paid to telemetry results, changes in cardiac enzymes, echocardiogram, ischemic stress test, and angiography results. Patients who could have benefited from coronary CTA screening without any need for subsequent hospital admission were identified. The cost implications of using a coronary CTA to triage patients in the ED compared with the current standard of care were assessed. Information about the average cost for patients hospitalized for ACP, ED costs, and the proposed cost of providing coronary CTA imaging studies were obtained from the hospital accounting department. The chi-square test was used to determine if the number of patients who would have benefited from coronary CTA screening within the period under review is significant. Descriptive analyses including pie charts and bar charts were used to compare associated costs. A flow chart showing the distribution of patients considered for this study is shown in Figure 1.

## Results

**Figure 2:**
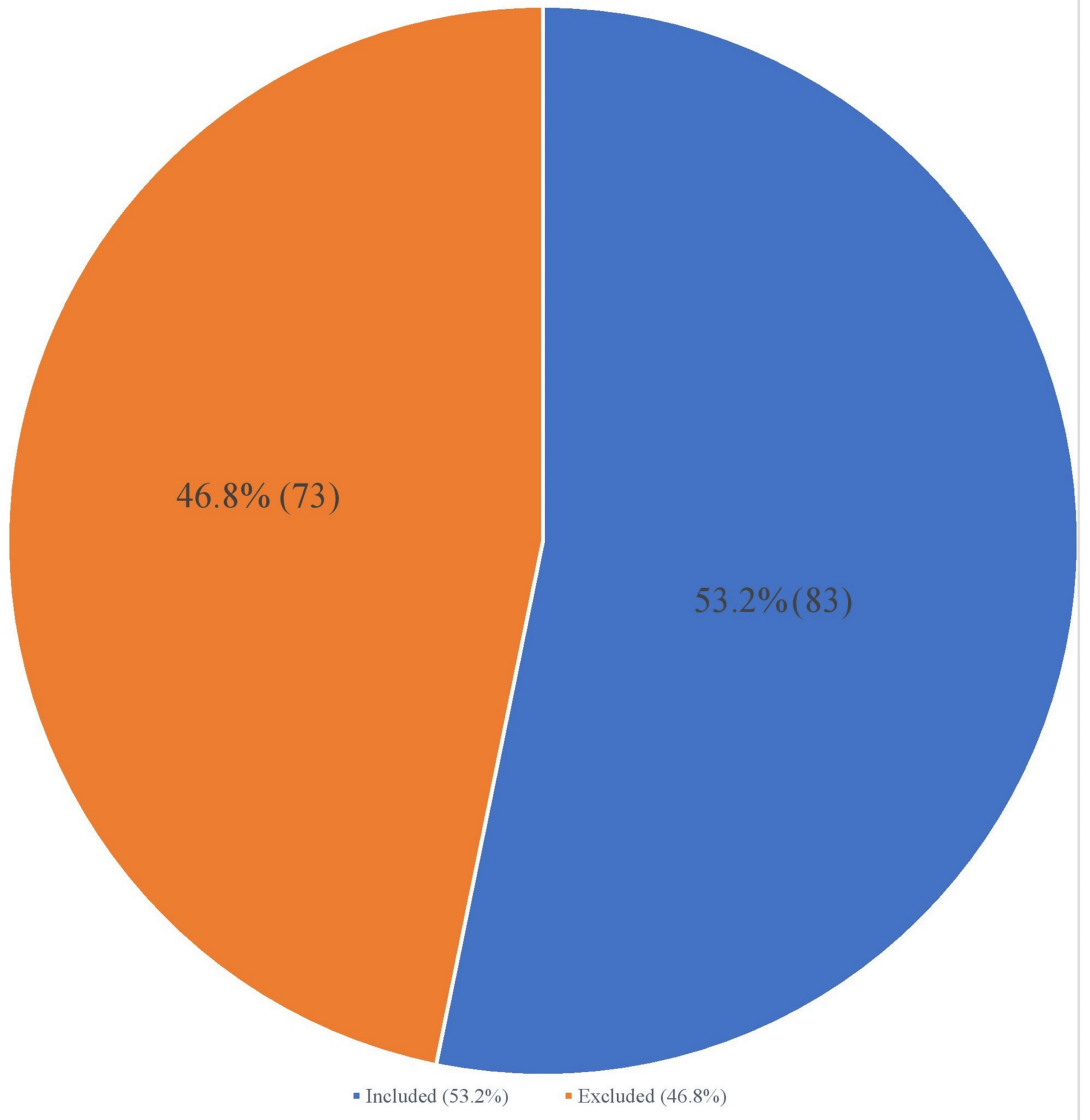
Overall distribution of patients admitted into the hospital for chest pain between July 2022 and June 2023

The total number of patients considered for this study was 156 out of which 73 patients were excluded based on already described criteria. Excluded patients are high-risk patients who need various levels of intervention and inpatient monitoring based on their medical history (stent, CABG, EKG abnormalities, elevated troponin, etc.). Only 83 patients fulfilled the inclusion criteria. These are patients with low to intermediate risk for CAD who were admitted into the hospital to rule out ACS within the period under review. In sum, 53.2% of patients admitted for chest pain met the inclusion criteria for the study while 46.8% were excluded (Figure 2).

**Figure 3:**
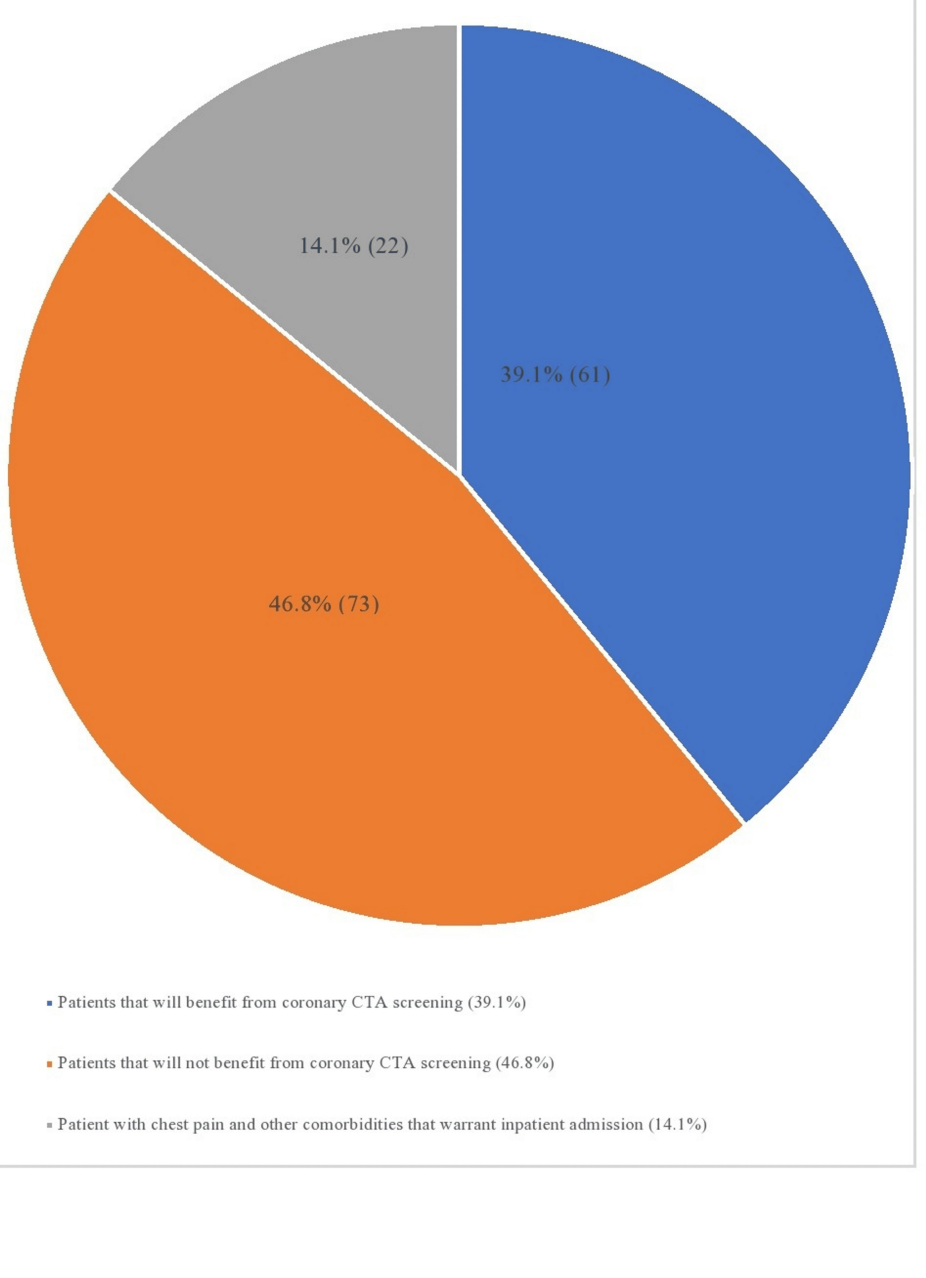
Further breakdown showing patients who would have benefited from coronary CTA screening CTA: Computed tomography angiography

Careful examination of the pool of patients included in the study revealed that 22 out of the total 83 patients who met the inclusion criteria would not have been discharged from the ED even if coronary CTA results were negative because they presented with other acute conditions that warranted inpatient management along with the ongoing chest pain. These conditions include arrhythmia, COPD exacerbation, asthma exacerbation, CHF exacerbation, pulmonary embolism, hypertensive emergency, etc. Consequently, only 61 patients would have benefited from coronary CTA screening consideration while 95 patients would not have benefited (Figure 3).

To put this in perspective, patients who would not have benefited from coronary CTA screening include a total of excluded patients based on established criteria (73 patients) and some patients who must be admitted into the hospital (despite fulfilling the inclusion criteria) due to other reasons regardless of the outcome of the coronary CTA screening (22 patients). Among patients finally included in the study, 33 underwent stress tests with one patient positive for reversible ischemia (subsequent angiography was negative). The average hospital stay was 2.3 days and the average age was 58.8 years old. There were 41 male patients and 42 female patients in the study.

Results showed that only 39.1% of patients admitted for chest pain during this period would have benefited from the use of coronary CTA for screening. While this result revealed that most patients admitted into the acute care facility for chest pain are high-risk patients with several comorbidities, the number of patients who could have benefitted from screening using the coronary CTA is statistically significant (P<0.001, Table 1).

**Table 1:** Chi-square statistical presentation showing the significance of the number of patients who would have benefited from coronary CTA screening *Significant CTA: Computed tomography angiography

	Patients not benefiting from screening		Patients benefiting from screening		Degree of freedom (df)	X^2^	P-Value
	N	%	N	%			
Coronary CTA screening	95	60.9	61	39.1	1	64.319	0.001*

**Figure 4:**
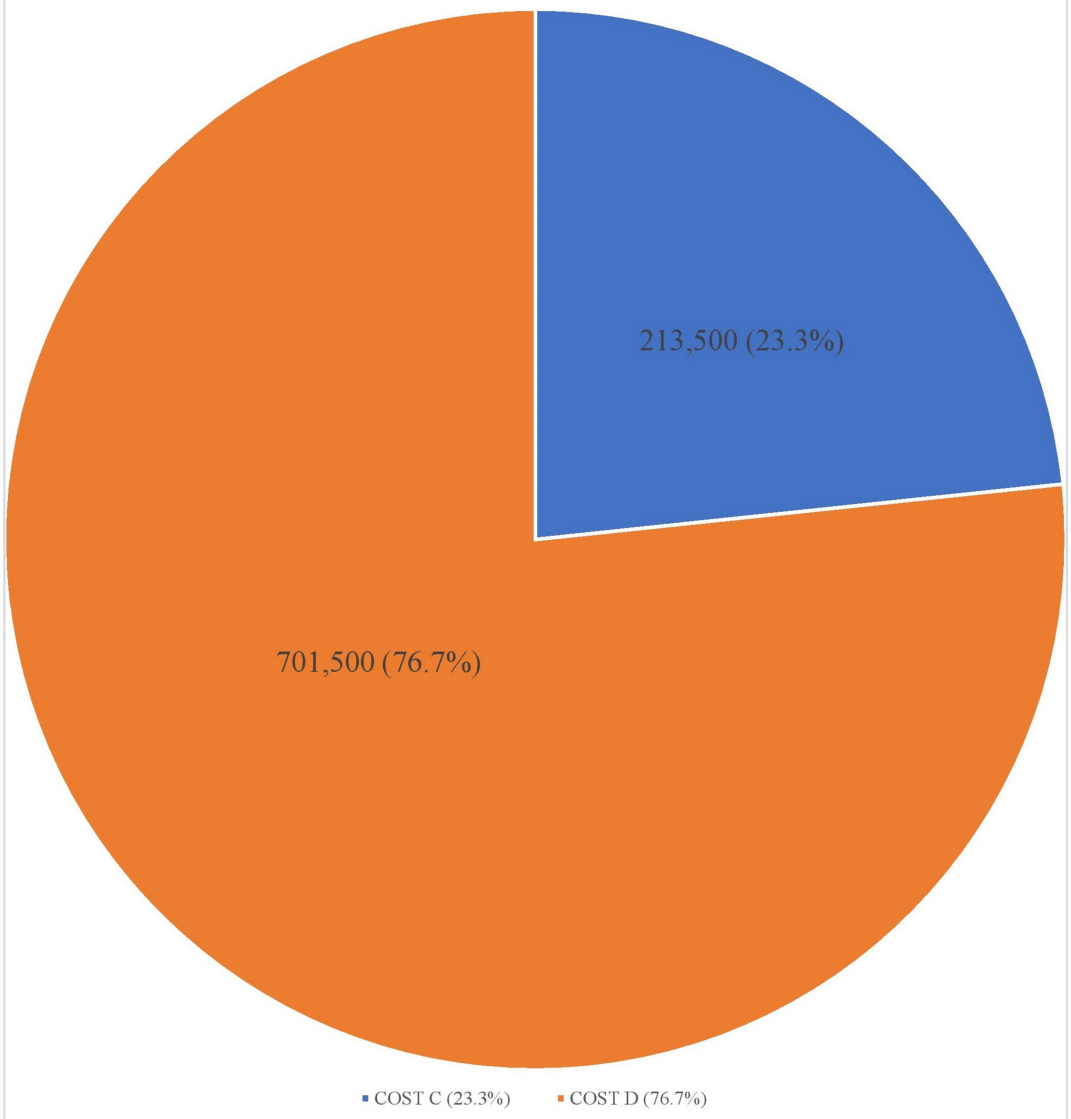
Cost that would have been saved if CTA was used in the ED CTA: Computed tomography angiography

Furthermore, the average cost of hospitalization for ACP management in the facility is $15,000. Therefore, the total cost for patients admitted for ACP between July 2022 and June 2023 was $2,340,000 (Cost A). Total inpatient cost incurred for patients eligible for coronary CTA was $915,000 (Cost B). Meanwhile, if these patients were screened with coronary CTA and discharged from the ED, the total cost incurred would have been $213,500 (Cost C) given the average cost of ED service at $500 and the average cost of coronary CTA screening at $3,000. Therefore, a total of $701,500 (76.7% of the hospitalization cost) would have been saved in healthcare costs if eligible patients were screened with coronary CTA and discharged from the ED (Figure 4).

**Figure 5:**
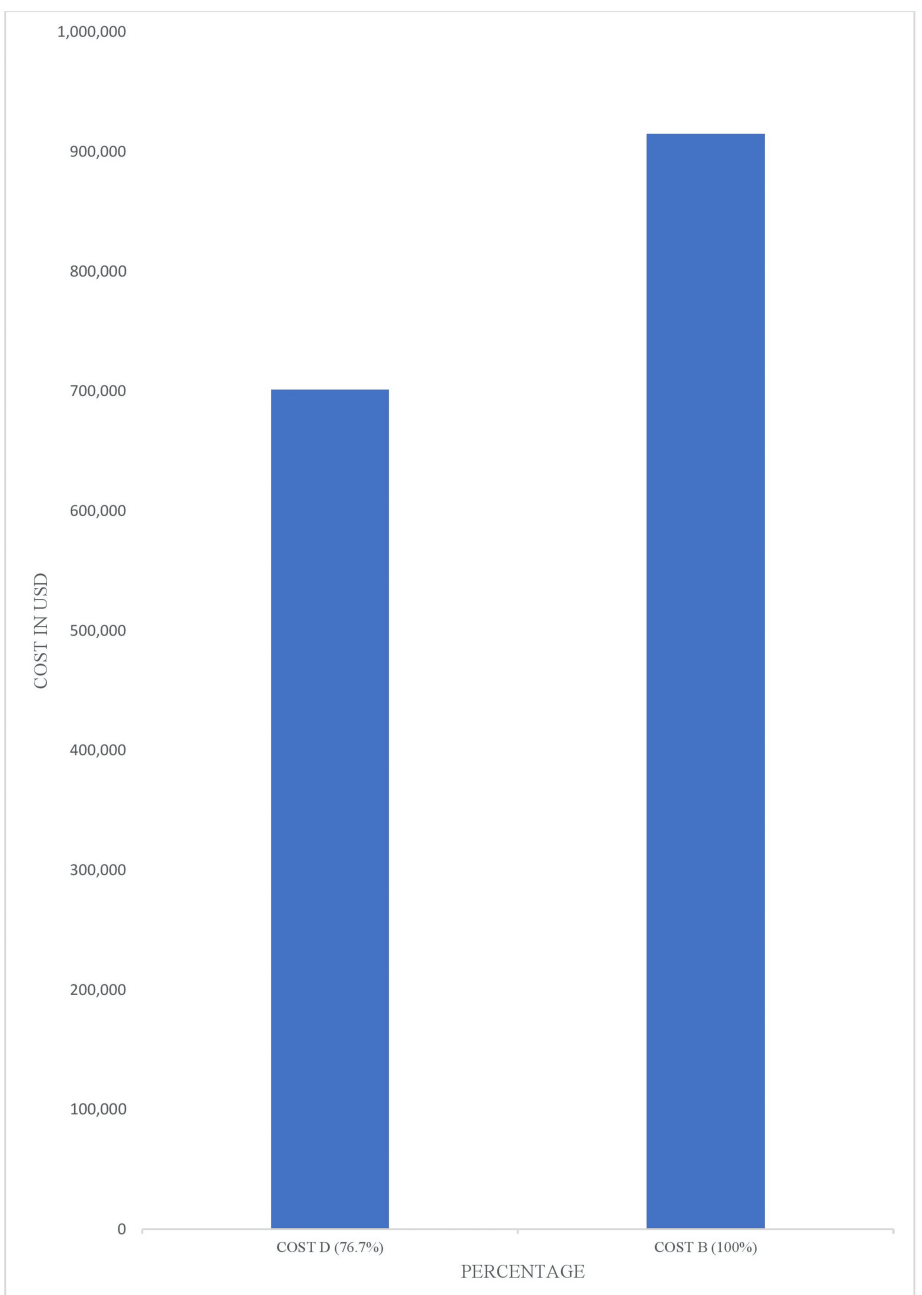
Comparison between the amount that would have been saved and the total cost incurred on inpatient services

To further corroborate this position, Cost D is substantial when compared with the total inpatient cost incurred (Cost B) as shown in Figure 5.

**Figure 6:**
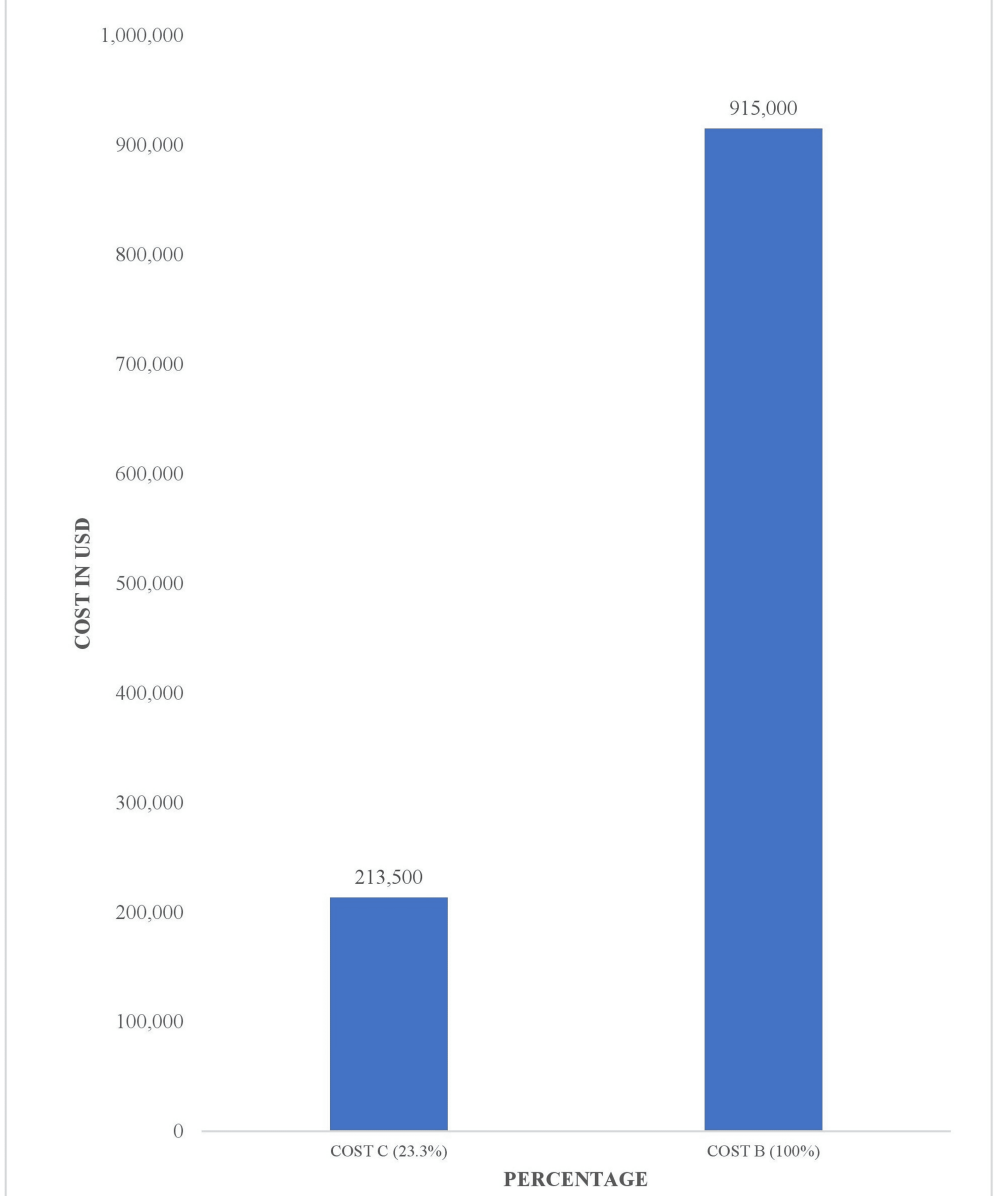
Cost of coronary CTA as a fraction of the total cost incurred on inpatient services CTA: Computed tomography angiography

However, Cost C is almost one-quarter of the total cost incurred for admitting patients eligible for coronary CTA screening (Figure 6).

Further details on cost analysis are highlighted below.

Cost A = total cost incurred for admitting all the patients with chest pain into the hospital in the period under review =156 (total number of patients) x $15,000 (average cost associated with ACP inpatient management in the hospital) = $2,340,000

Cost B = Cost associated with hospitalization of patients that would have been eligible for coronary CTA = 61 (total number of eligible patients for coronary CTA) x $15,000 = $915,000

Cost C = Costs that would have been expended if patients eligible for coronary CTA were screened in the ED and discharged = $500 (average cost of ED service) x61 patients + $3,000 (average cost of coronary CTA) x61 patients = $213,500

Cost D = Amount that would have been saved if patients eligible for coronary CTA were not hospitalized = Cost B - Cost C = $915,000 - $213,500 = $701,500 (76.6% of cost B).

## Discussion

Since CADs contribute significantly to mortality and morbidity in this century, continuous efforts are being directed toward timely and efficient diagnosis [9]. While different modalities are used for assessing patients, coronary CTA has been identified as a preferred and cost-effective option for evaluating coronary arteries [10]. It is a rapid non-invasive test that can effectively exclude CADs by assessing the anatomy of coronary arteries. In addition to locating obstructive CADs, coronary CTA can also help diagnose non-obstructive CAD and thereby guide ASCVD risk-lowering initiatives to improve clinical outcomes [11]. It is against this backdrop that coronary CTA has been identified as a “gatekeeper” diagnostic test prior to the use of invasive angiography [12]. In fact, some randomized trials have shown that the use of coronary CTA to triage patients in the ED was associated with decreased hospital admissions and reduced length of hospital stay [13]. Having established the effectiveness of coronary CTA in the triage of patients with low to intermediate risk for CAD, it is also important to consider its long-term utility as a cost-effective option in this regard. The potential of coronary CTA to significantly reduce the economic burden associated with the diagnosis and treatment of CAD by providing a cost-effective diagnostic tool was established in a review [14]. In fact, for patients with positive stress test results without symptoms, performing coronary CTA before cardiac catheterization has been established as a cost-effective strategy when the probability of patients having significant CAD is less than 50% [15]. Coronary CTA has the potential to reduce healthcare costs in different ways. Firstly, it can lower the number of unnecessary hospital admissions and subsequent exposure to hospital-associated infections (HAIs). Financial and economic burden of HAIs on our healthcare system cannot be overemphasized. It compromises patients’ health and stretches healthcare resources unnecessarily. Therefore, the drive to reduce unnecessary admissions to the hospital is beneficial to the patients and the system. Furthermore, since coronary CTA accurately identifies CAD patients who need further work-up, it helps to channel healthcare resources in a more effective way. This approach helps to conserve healthcare resources and minimize iatrogenic harm. Similarly, the cost associated with screening CAD patients with coronary CTA in the ED is lower when compared with the cost of hospital admission and in-patient work-up. Patients with negative coronary CTA can be safely discharged from the ED thereby lowering the economic burden of unnecessary tests and hospital stay. Results of this study have shown that lots of money would have been saved if coronary CTA was used in this facility to triage patients with low to intermediate risk for CAD.

Meanwhile, this study has some limitations. To start with, there are patient-related medical issues that could make coronary CTA screening impractical. Some of these drawbacks include obesity, arrhythmia, pregnancy, kidney disease, contrast allergies, and contraindications to beta-blockers or nitroglycerin use. If the number of patients having these constraints is significant in any acute care facility, the cost-benefit of deploying coronary CTA may not be profound. Also, image quality could be affected by motion artifacts and calcified plaques. This may grossly limit the diagnostic yield of coronary CTA and eliminate its advantages over the current standard of care. Furthermore, our cost analysis is based on specific information provided by the acute care facility. Costs associated with services in other hospitals may be different thereby limiting the generalizability of our findings. Also, costs associated with the acquisition of coronary CTA along with depreciation costs and other ancillary expenses incurred while operating the equipment were not included in the analysis. In addition, one-year data was considered and analyzed. This may have some effects on the power of our study.

## Conclusions

Our study revealed that the number of patients with low to intermediate risk for chest pain admitted to the acute care facility between July 2022 and June 2023 is statistically significant and can justify an investment in coronary CTA. Also, cost analysis revealed that lots of money would have been saved if qualified patients were screened with coronary CTA and discharged from the ED contrary to the current practice of admitting everyone into the hospital to “rule out ACS.” Therefore, considering our patients’ pool which is largely based on the community where the hospital is located, investment in coronary CTA is justified and will contribute to lowering healthcare costs. To further increase the power of the study, a similar investigation comprising patients admitted to the hospital for ACP over a five-year window is suggested. Also, a full comprehensive cost analysis model can be considered.
